# Milrinone and levosimendan improve microvascular perfusion in septic rats: a randomized, placebo-controlled trial

**DOI:** 10.1186/s40635-026-00881-w

**Published:** 2026-03-06

**Authors:** Carsten Marcus, Stefan Hof, Alena Gesing, Philisa Thelen, Sarah Orzol, Antonia Vocke, Jan Schulz, Anne Konstanze Charlotte Kuebart, Richard Truse, Christian Vollmer, Inge Bauer, Olaf Picker, Anna Herminghaus

**Affiliations:** 1 Department of Anesthesiology, University Hospital Duesseldorf, Moorenstrasse 5, 40225 Duesseldorf, Germany; 2 Department of Anesthesia, Critical Care and Pain Medicine, Beth Israel Deaconess Medical Center, Harvard Medical School, 330 Brookline Avenue, Boston, MA 02215 USA

**Keywords:** Sepsis, Microcirculation, Inotropic agents, Vasopressin, Mitochondria, Colon, Liver, CASP, Animal model

## Abstract

**Background:**

Microcirculatory dysfunction is a key pathophysiological feature of sepsis and contributes to organ failure and mortality. In the gastrointestinal tract, impaired barrier function due to microcirculatory injury promotes translocation of inflammatory mediators and bacteria, worsening systemic inflammation and multiorgan dysfunction. Inotropic and vasoactive agents may improve microvascular perfusion through vasodilation in addition to their inotropic effects. Milrinone, a phosphodiesterase-3 inhibitor, and levosimendan, a calcium sensitizer, have shown promising but inconsistent effects in sepsis, while data on their direct microcirculatory and mitochondrial effects in abdominal organs remain limited. Sub-therapeutic vasopressin has demonstrated beneficial effects on gut microcirculation in experimental models, but its combination with inotropes has not been investigated. We hypothesized that (1) milrinone and levosimendan increase colonic and hepatic microvascular blood flow and oxygenation, (2) adjunctive low-dose vasopressin further enhances gastrointestinal microcirculation, and (3) mitochondrial respiration does not differ between treatment groups.

**Methods:**

Male Wistar rats (*n* = 105) underwent colon ascendens stent peritonitis (CASP) or sham surgery to induce moderate sepsis. Twenty-four hours later, animals received intravenous infusions of vehicle, milrinone, levosimendan, or the respective inotrope with low-dose vasopressin. Colonic and hepatic microvascular oxygenation and blood flow were assessed using tissue-reflectance spectrophotometry and laser Doppler flowmetry. Mitochondrial respiration in colonic and hepatic tissue homogenates from septic animals was analyzed by respirometry. Statistical analyses included mixed-effects models with Tukey or Dunnett post-hoc tests and Kruskal–Wallis tests with Dunn’s correction, using a two-sided significance level of *α* = 0.05.

**Results:**

In septic animals, milrinone and levosimendan increased colonic and hepatic microvascular blood flow. With adjunctive vasopressin, colonic perfusion remained increased, whereas hepatic blood flow did not increase. Microvascular oxygenation remained unchanged in both organs. In sham-operated animals, microvascular blood flow and oxygenation did not differ between treatment groups. Mitochondrial respiration in colon and liver was unchanged across treatments, as indicated by respiratory control index and ADP/O ratio.

**Conclusions:**

In experimental abdominal sepsis, milrinone and levosimendan increase colonic and hepatic microvascular blood flow without affecting mitochondrial respiration. Adjunctive vasopressin alters hepatic but not colonic microvascular responses during combined inotropic therapy.

**Supplementary Information:**

The online version contains supplementary material available at 10.1186/s40635-026-00881-w.

## Background

Sepsis, one of the most prevalent problems in intensive and perioperative care medicine is defined as a life-threatening organ dysfunction caused by a dysregulated host response to infection [1]. It is accompanied by inflammation, cytokine production, and a multitude of pathophysiological changes throughout the organism. Taken in mind, that sepsis still causes annually over 11 million deaths worldwide, with a largely constant lethality over the last 20 years, its importance as a topic of research is undisputable [2]. Among others, the loss of gastrointestinal barrier function is one major determinant to overall morbidity in septic patients. As the intestinal mucosa is exposed to a complex variety of microorganisms and pathogens, impaired regional tissue integrity might lead to a translocation of bacteria and pathogen associated proteins into the systemic circulation [3, 4] and might engrave the global derangement in patients with sepsis and septic shock. Since the liver plays a pivotal role in the clearance of bacteria and endotoxins, as well as in the immunological and metabolic response to infections progressive hepatic failure has to be declared as another contributing factor in the development and progress of sepsis and septic shock [5, 6]. Importantly, neither intestinal nor hepatic function can be adequately replaced by mechanical organ support in the intensive care unit, making preventive strategies to preserve a physiological function of these both organs all the more essential.

A key hallmark of sepsis is impaired microcirculation followed by mitochondrial dysfunction after depletion of the regional oxygen reserve [7]. Whereas microvascular derangements and mitochondrial dysfunction were precursors of organ failure and increased mortality, early improvement of microvascular perfusion was associated with reduced multi-organ dysfunction [8]. Improving microvascular perfusion and mitochondrial respiration could therefore be a promising target to prevent critically ill patients from multi-organ dysfunction syndrome (MODS) leading to poor outcome and death.

An impairment of cardiac function, also called septic cardiomyopathy or sepsis-induced myocardial dysfunction, is observed in up to 50% of septic patients [9, 10] and could reduce microvascular tissue perfusion. Further, excessive vasoconstriction by endogenous or exogenous catecholamines might increase microvascular resistance and could reduce microvascular blood flow of the subordinated tissue. Therefore, some patients with septic shock could profit from a pharmacological treatment aiming to increase cardiac output and reduce systemic vascular resistance. In this context, our research group investigated the effect of dobutamine administration on intestinal and hepatic microcirculation. Although systemic administration of dobutamine has been revealed to be a promising therapeutic approach to improve microvascular perfusion and oxygenation in septic animals, this effect might be limited in prolonged shock states with excessive catecholamine release and internalization of β-adrenergic receptors [11, 12].

Taken these aspects into account, the current study aims to investigate the effect of milrinone, a phosphodiesterase-3-inhibitor, and levosimendan, a calcium sensitizer, as two inotropic agents that act independent of β-adrenergic receptor activation, on intestinal and hepatic microvascular blood flow and oxygenation in healthy and septic rats. Because of the different mode of action, these agents are promising therapeutic agents to improve microvascular blood flow and oxygenation in dobutamine refractory, sepsis-induced myocardial dysfunction. However, studies investigating the effect of milrinone and levosimendan in septic patients led to contradictory results and usually do not consider microvascular effects of milrinone and levosimendan. More in detail, an experimental study in septic mice reported lower concentrations of myocardial injury after systemic milrinone and levosimendan treatment [13]. Further, a retrospective study in septic patients revealed improved cardiac index and central venous oxygen saturation in patients receiving milrinone [14]. Contradictory to these studies presuming a beneficial effect of milrinone on cardiac failure during sepsis, a large retrospective analysis on patients with sepsis could not show a significant association between milrinone therapy and reduced organ dysfunction or mortality [15].

Different from milrinone application, that results in higher cyclic adenosine monophosphate levels and increased myocardial calcium concentration, levosimendan is a drug in the class of calcium sensitizers, which increases the effect of calcium on the myocardial, contractile proteins. With a focus on septic cardiomyopathy, an experimental study in rats investigating the effects of levosimendan on myocardial tissue injury reported less damage to the rodent myocardium in septic animals after levosimendan treatment. Further, levosimendan enhanced ventricular function in septic patients with cardiomyopathy who were unresponsive to dobutamine [13, 16]. However, despite this promising results the LEOPARDS trial, a double-blinded, randomized controlled study including over 500 septic patients could not show any decrease in mortality [17].

Most of the mentioned studies focused on hemodynamic parameters, serum biomarkers or clinical outcomes like mortality and Sequential Organ Failure Assessment (SOFA)-Score, whereas data on microcirculation itself remains scarce. One study in six non-septic foxhounds showed a beneficial effect of levosimendan on mucosal hemoglobin oxygenation [18]. For milrinone, one study reported positive effects of after administration in septic hamsters, including attenuation of endotoxemia-induced arteriolar vasoconstriction and capillary perfusion deficits (improved functional capillary density and capillary red blood cell velocity) [19]. It seems plausible that, besides their inotropic effects, the vasodilatory properties of these drugs could positively influence microvascular blood flow and tissue oxygenation.

Whereas inotropic agents aim to increase microvascular perfusion by a quantitative turnover of the circulating blood, vasoactive agents such as norepinephrine or vasopressin are commonly used to stabilize systemic blood pressure in septic patients. However, higher doses might impair microvascular perfusion by excessive vasoconstriction, reduced regional oxygen supply to the subordinated tissue and promote intestinal hypoxia [20]. It becomes clear, that the administration of vasoconstrictive agents should not be guided solely by systemic blood pressure and that the use of vasoactive agents should be continued to be discussed [21]. Arginine-vasopressin, a V_1_-receptor agonist, is widely used to treat hypotension in septic shock and revealed beneficial effects on gastrointestinal microcirculation when used in sub-therapeutic doses in preclinical animal studies. More detailed, Truse et al. reported a dose-dependent effect of exogenous vasopressin application on gastric microvascular oxygenation in female dogs under physiological conditions. In this study, sub-therapeutic doses of exogenous vasopressin increased gastric microvascular oxygenation without exerting a significant effect on systemic hemodynamic variables, whereas higher vasopressin doses led to decreased values of microvascular oxygenation. In accordance, Schulz et al. revealed a beneficial effect of sub-therapeutic but not therapeutic vasopressin application on intestinal microvascular oxygenation in rats with abdominal sepsis and inflammation [22, 23]. Taken these findings together, titrated vasopressin application might improve gastrointestinal tissue oxygenation independent of macrocirculatory variables and serve as an additional pharmacological strategy to improve colonic and hepatic microvascular perfusion and tissue integrity. To broaden the therapeutic options for microvascular dysfunction and subsequently improve tissue oxygenation in septic patients, we also examined the impact of an adjunctive arginine-vasopressin application on microvascular perfusion and tissue oxygenation in the recent study.

As impaired intestinal microcirculation might lead to mitochondrial dysfunction we not only focused on microvascular effects of the investigated agents but also assessed their impact on mitochondrial respiration in homogenates of the colon and the liver of the septic animals. Building on these considerations, we designed a randomized, placebo-controlled experimental study to investigate the following hypotheses:

1. The inotropic agents milrinone and levosimendan increase colonic and hepatic microvascular blood flow, leading to improved microvascular oxygenation in septic rats.
2. An additional treatment with sub-therapeutic vasopressin further enhances microcirculation in colon and liver.
3. Since the beneficial effects of milrinone, levosimendan, and adjunctive vasopressin application on microvascular tissue oxygenation are primarily mediated by an optimization of regional microcirculation, mitochondrial respiration does not differ between the experimental groups in septic animals.

## Material and methods

### Induction of sepsis

All parts of this study were performed in accordance with NIH guidelines for animal care and reported in accordance with the ARRIVE guidelines and the MQTiPSS recommendations for preclinical sepsis research [24, 25]. Experiments started after approval from the local Animal Care and Use Committee (Landesamt fuer Natur, Umwelt und Verbraucherschutz, Recklinghausen, Germany, Az. 81–02.04.2020.A313).

**Fig. 1:**
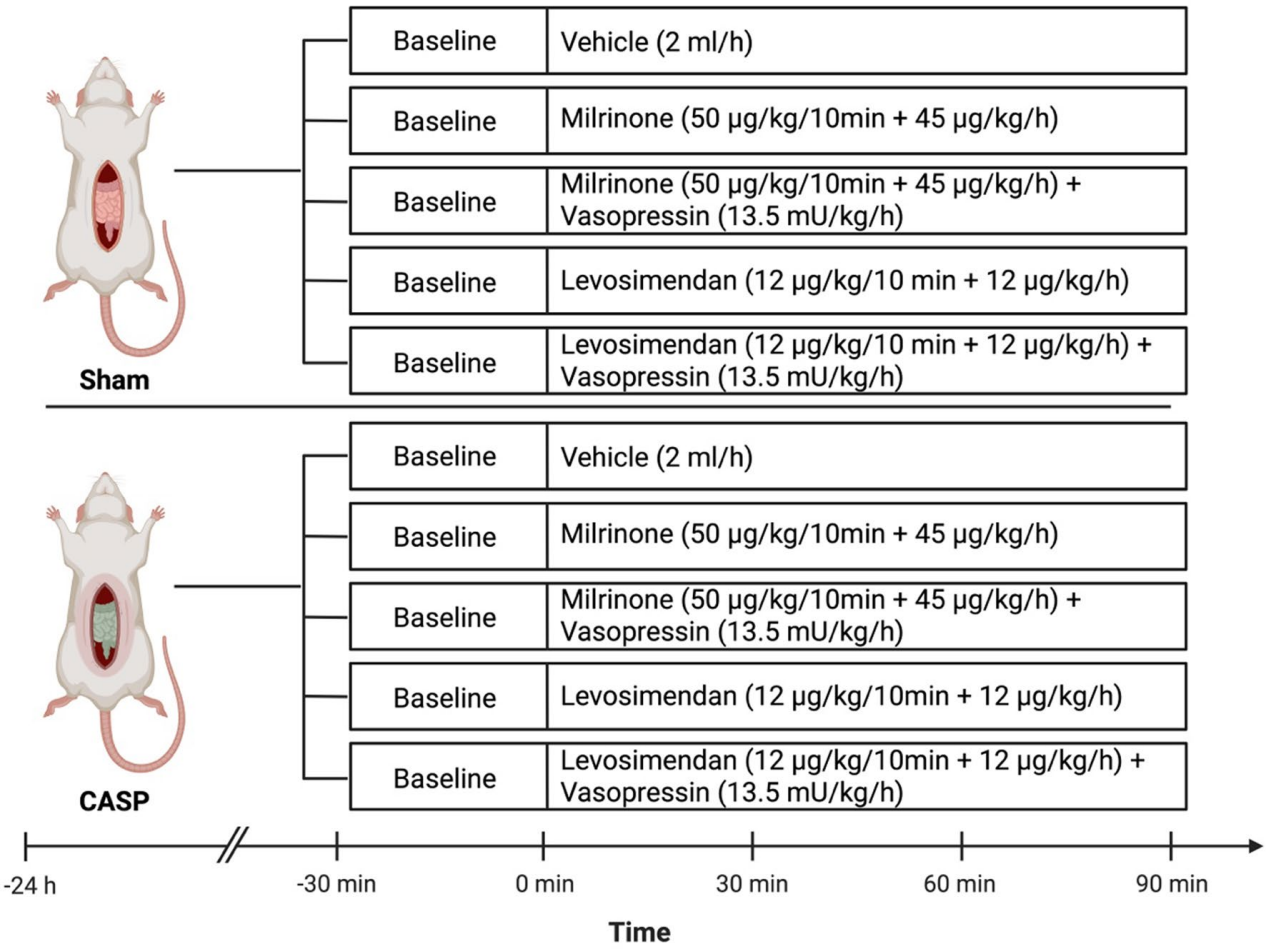
Experimental protocol. Colon ascendens stent peritonitis (CASP) or sham surgery was performed 24 h before microcirculatory measurements, Created with BioRender.com

The entire experiment was completed in 105 male Wistar rats (315–385 g), which were provided by the breeding facility at the Central Animal Research Facility of the Heinrich-Heine-University Duesseldorf. The animals were randomly assigned to 10 groups, of which 5 underwent a CASP (Colon ascendens stent peritonitis) surgery to create abdominal sepsis and 5 underwent a sham surgery to serve as control group. Animals were treated with a balanced crystalloid solution (vehicle), milrinone, levosimendan, milrinone + vasopressin or levosimendan + vasopressin (Fig. 1). Final group sizes varied due to technical issues during data acquisition and are reported in Supplementary Table (S3).

The experiments were carried out in the laboratory of the Department of Anesthesiology, University Hospital Duesseldorf, Germany. CASP and sham surgery was done as described earlier [26]. Briefly, a median laparotomy (2–3 cm) was performed under general anesthesia (Buprenorphine 0.1 mg·kg⁻^1^, subcutaneous (s.c.); Eumedica Pharmaceuticals GmbH, Lörrach, Germany. Sevoflurane 3.0–3.2% inhalative FiO₂ 0.5; AbbVie Deutschland GmbH & Co KG, Ludwigshafen, Germany) [27, 28]. Abdominal sepsis was induced using a modified CASP technique: two 14 G stents were inserted into the colon 1 cm distal to the ileocecal valve and secured to allow continuous leakage of fecal content into the peritoneal cavity [29]. The peritoneum was closed with a running suture and the skin with single interrupted stitches. In sham-operated animals, the abdomen was opened similarly, but a single stent was fixed to the colonic wall without perforation [27].

After sham or CASP surgery, the animals were kept in individual cages (Makrolon cage Typ III, surface area 825 cm^2^, height 15 cm, Ehret GmbH, Emmendingen, Germany), and received sufficient analgesia with buprenorphine (0.1 mg · kg^−1^, s.c.) every 8 h. Access to water and food ad libitum was provided as well as a 12 h day and night rhythm and a constant temperature (24 ± 2 ℃) and humidity (50 ± 5%). Every 6 h the animals were scored using the earlier described Septic Rat Severity Score (SRSS-System), a standardized protocol to determine the severity of sepsis and to monitor the animals welfare [22]. The scoring of all animals was performed by a registered veterinary. If the score was 10 or more, the animal was euthanised with pentobarbital (600 mg · kg^−1^, intraperitoneal (i.p.); Boehringer Ingelheim Vetmedica GmbH, Ingelheim Germany).

### Assessment of microcirculation

Twenty-four hours after the CASP or sham operation, the animals underwent general anesthesia with a subcutaneous injection of buprenorphine (0.1 mg · kg⁻^1^) and an i.p. injection of sodium pentobarbital (60 mg · kg⁻^1^). Placed on a heating pad, the animals were tracheotomized and volume-controlled, pressure-limited mechanical ventilation was initiated (70 min⁻^1^, VT 1.8–2.5 mL, PAW < 17 cmH_2_O, FiO_2_ 0.3, FiN₂ 0.7) using the Inspira Advanced Safety Ventilator (Harvard Apparatus GmbH, March-Hugstetten, Germany). Carotid artery and jugular vein were cannulated with 24 G catheters for invasive blood pressure monitoring, arterial blood gas sampling, and drug/infusion administration. Following the establishment of a vascular access, all animals received an intravenous application of piperacillin/tazobactam (120 mg·kg⁻^1^; Fresenius Kabi, Bad Homburg, Germany) as antibiotic therapy. Arterial blood gases were obtained at baseline and every 30 min. Further, pentobarbital (10 mg·kg⁻^1^·h⁻^1^) was infused intravenously to maintain anesthesia.

For the microcirculatory measurements, the animals were relaparotomized and flexible light guide probes (O2C LW 2222, Lea Medizintechnik GmbH, Giessen, Germany) were placed on the liver surface as well as on the colon ascendens next to the stent to measure intestinal microcirculation [30]. Microvascular measurements were performed on the serosal surface to minimize confounding effects from local trauma and manipulation, such as those that would result from surgical opening of the intestine. In a large-animal model, serosal microcirculation has been shown to be comparable to mucosal measurements [31]. Colonic microvascular hemoglobin oxygen saturation (µHbO_2_) and perfusion (µFlow) were measured using tissue-reflectance spectrophotometry and laser Doppler flowmetry. This technique assesses oxygenation of the microvasculature (< 100 µm) and calculates microvascular perfusion from erythrocyte velocity and concentration. Since the largest amount of microvascular erythrocytes is stored within the capillaries and the postcapillary veins the calculated variables predominantly represent microvascular conditions. Signal quality was continuously monitored, and values were averaged over the last 5 min of each 30-min interval.

After a 30-min baseline recording, animals received intravenous therapy for 90 min with either the crystalloid vehicle (2 mL·h⁻^1^; Fresenius Kabi AG, Bad Homburg, Germany), milrinone (50 µg·kg⁻^1^ over 10 min followed by 45 µg·kg⁻^1^·h⁻^1^; Hikma Pharma GmbH, Planegg, Germany), milrinone combined with vasopressin (13.5 mU·kg⁻^1^·h⁻^1^; Amomed Pharma GmbH, Vienna, Austria), levosimendan (12 µg·kg⁻^1^ over 10 min followed by 12 µg·kg⁻^1^·h⁻^1^; Carinopharm GmbH, Eime, Germany), or levosimendan combined with vasopressin. The vasopressin dose corresponds to a fraction of clinically used vasopressor doses in septic shock (27–34 mU·kg⁻^1^·h⁻^1^), and was deliberately selected as a sub-therapeutic, non-pressor dose, based on prior trials [22]. Dosing regimens for milrinone and levosimendan were chosen according to clinically used doses and supported by experimental studies demonstrating protective effects on the intestinal microcirculation [22, 33, 34]. Before application, drugs were diluted in the vehicle solution to ensure equal volume administration in all groups (2 mL·h⁻^1^). All investigators were blinded to the pharmacological treatments. At the end of the experiments, all animals were euthanized under deep anesthesia by exsanguination at the end of the experiments. Colon and liver tissues were retrieved post-mortem from the septic rats.

### Mitochondrial measurements in septic rats

To specifically assess sepsis-induced alterations in mitochondrial function, mitochondrial measurements were performed exclusively in septic animals. Colon and liver homogenates were prepared as previously described [35, 36]. Freshly harvested colonic tissue was placed in ice-cold isolation buffer (200 mM mannitol, 50 mM sucrose, 5 mM KH₂PO₄, 5 mM morpholinepropanesulfonic acid (MOPS), 1 mM EDTA, pH 7.15). The colon was then opened, cleaned and treated with trypsin on ice for 5 min. The samples were then transferred to isolation buffer containing 20 mg·ml^−1^ (2%) BSA and protease inhibitors (cOmplete^™^ Protease Inhibitor Cocktail, Roche Life Science, Mannheim, Germany), cut into 2–3 mm^3^ pieces, and homogenized using a Potter–Elvehjem homogenizer (5 strokes, 2000 rpm). Liver tissue was processed similarly: freshly harvested liver was directly placed in ice-cold isolation buffer, cut into 2–3 mm^3^ pieces and homogenized. Protein concentrations in the tissue homogenates were determined using the Lowry method with bovine serum albumin as a standard [37].

Mitochondrial oxygen consumption was measured at 30 °C using a Clark electrode (model 782, Strathkelvin Instruments, Glasgow, Scotland) [30, 35]. Tissue homogenates were suspended in respiration medium (130 mM KCl, 5 mM K₂HPO₄, 20 mM MOPS, 2.5 mM EGTA, 1 μM Na₄P₂O₇, 0.1% BSA for liver and 2% BSA for colon, pH 7.15) to achieve a protein concentration of 6 mg·ml^−1^ for colon and 4 mg·ml^−1^ for liver samples. Mitochondrial state 2 respiration was recorded in the presence of either complex I substrates (2.5 mM glutamate and 2.5 mM malate, G-M) or the complex II substrate (5 mM succinate for colon, 10 mM for liver) in combination with the complex I inhibitor rotenone (0.5 μM) to prevent complex I activation. Maximal mitochondrial respiration in state 3 was measured following the addition of 50 μM ADP for colon and 250 µM for the liver tissue. The respiratory control index (RCI), which is defined as the ratio of state 3 to state 2 respiration, was calculated to assess the coupling between the electron transport chain and oxidative phosphorylation. Additionally, the ADP/O ratio was determined by comparing the amount of ADP added to the oxygen consumption, providing insight into the efficiency of oxidative phosphorylation. Oxygen consumption values represent the mean of three technical replicates. The solubility of oxygen was assumed to be 223 μmol O₂·l⁻^1^ at 30 °C, as stated in the Strathkelvin Instruments manual. Respiration rates are expressed in nmol O₂·min^−1^·mg^−1^ protein. Mitochondrial membrane integrity was assessed by adding 2.5 μM cytochrome c and 0.05 μg·ml^−1^ oligomycin. The absence of an increase in respiration upon cytochrome c addition confirmed the integrity of the outer mitochondrial membrane. Inhibition of adenosine triphosphate (ATP) synthesis by oligomycin shifted the mitochondria to state 2, reflecting the respiration rate required to compensate for proton leakage. These findings indicate that the inner mitochondrial membrane remained intact and that the mitochondria were not damaged during the preparation process.

### Statistical analysis

The a priori power analysis (G*Power Version 3.1.7, University of Duesseldorf, Duesseldorf, Germany) with *n* = 12 animals per group at a given *α* ≤ 0.05 (two-tailed) and an expected difference in µHbO_2_ and µFlow of at least 20% with an expected standard deviation of 10% to 15% (based on previous studies) suggested a power of 84.5%. In accordance with the 3R principle, the trials were just performed in *n* = 10 animals in the respective sham-operated groups, accepting a corresponding reduction in statistical power.

Microvascular oxygenation (µHbO_2_) and microvascular perfusion (µFlow) were analyzed with a mixed effects model, followed by Tukey post-hoc test for differences between groups, and Dunnett post-hoc test for differences versus baseline. Mitochondrial data were analyzed with Kruskal–Wallis’-test with Dunn’s post hoc correction for multiple comparisons. All statistical tests were performed via GraphPad software v 10.1., Int, La Jolla, CA. Microcirculatory data are expressed as means ± SD, while mitochondrial data are presented as box plots (min/median/max). A *p*-value of < 0.05 was considered statistically significant. Baseline values were normalized and set to zero, Delta values (Δ) were calculated by subtracting the absolute baseline value from the corresponding value after 30, 60, and 90 min. Accordingly, both microvascular oxygenation (ΔµHbO_2_) and microvascular perfusion (ΔµFlow) are reported as absolute differences from the respective individual baseline values, expressed in percentage points [%] and arbitrary units [AU]. To reduce complexity and redundancy in the main text and figures, all descriptive data (mean ± SD and number of animals per group) for HR, MAP, µHbO₂ and µFlow in colon and liver are provided in the supplementary material (Supplemental tables S3–S5).

## Results

### Macrocirculation

**Fig. 2:**
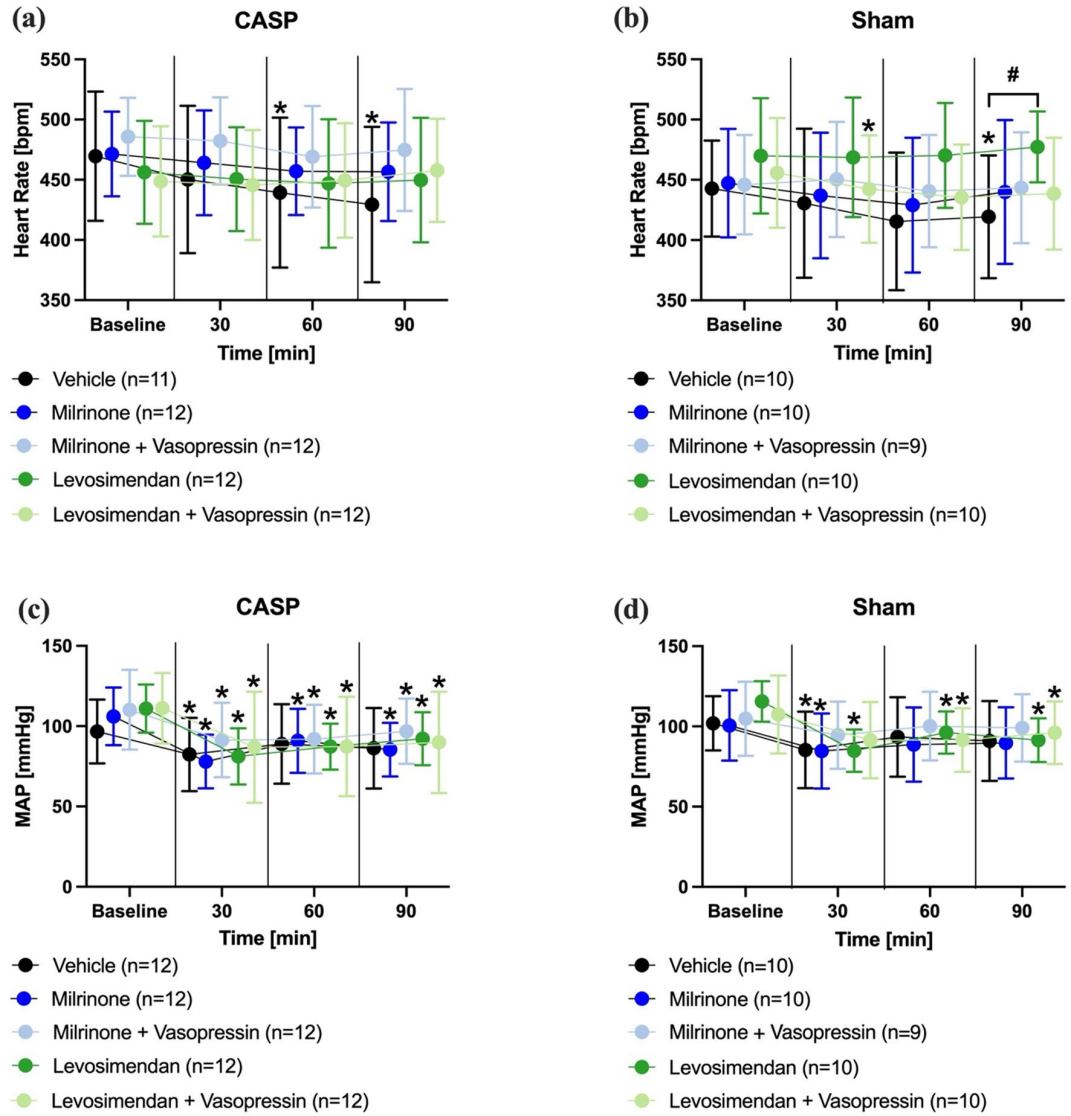
Heart rate and mean arterial pressure. Effect of inotrope therapy and vasopressin on heart rate ([bpm]; **a**, **b**) and mean arterial pressure (MAP [mmHg]; **b**, **c**) in colon ascendens stent peritonitis (CASP) or sham operated animals, data are presented as mean ± SD, *n* = 9–12 per group, * = *p* < 0.05 vs. baseline, # = *p* < 0.05 vs. vehicle (mixed-effects models with Dunnett/Tukey post hoc correction)

Heart rate decreased in the sepsis-control group (Fig. 2a) as well as in the sham-operated animals without pharmacological treatment (Fig. 2b) over time. In animals that received a pharmacological treatment with levosimendan, milrinone or a combination of one inotropic agent and vasopressin heart rate remained unchanged. This finding was independent of the surgical pretreatment. The heart rate in sham-operated animals treated with levosimendan was significantly higher than in the respective control group at the same point of time. The combination of levosimendan and vasopressin significantly decreased the heart rate after 30 min (Fig. 2b).

Mean arterial pressure decreased over time in septic animals independent of the respective pharmacological treatment after 30 min. Additionally, arterial blood pressure remained depressed in septic animals that received milrinone, levosimendan or a combination with adjunct vasopressin application (Fig. 2c). In sham-operated animals, arterial blood pressure decreased after 30 min, whereas the levosimendan and levosimendan + vasopressin group showed a decrease in MAP after 30, 60 and 90 min (60 and 90 for levosimendan and vasopressin). The sole application of milrinone led to a decrease in blood pressure after 30 min (Fig. 2d).

### Microvascular blood flow and tissue oxygenation

#### Colon

**Fig. 3:**
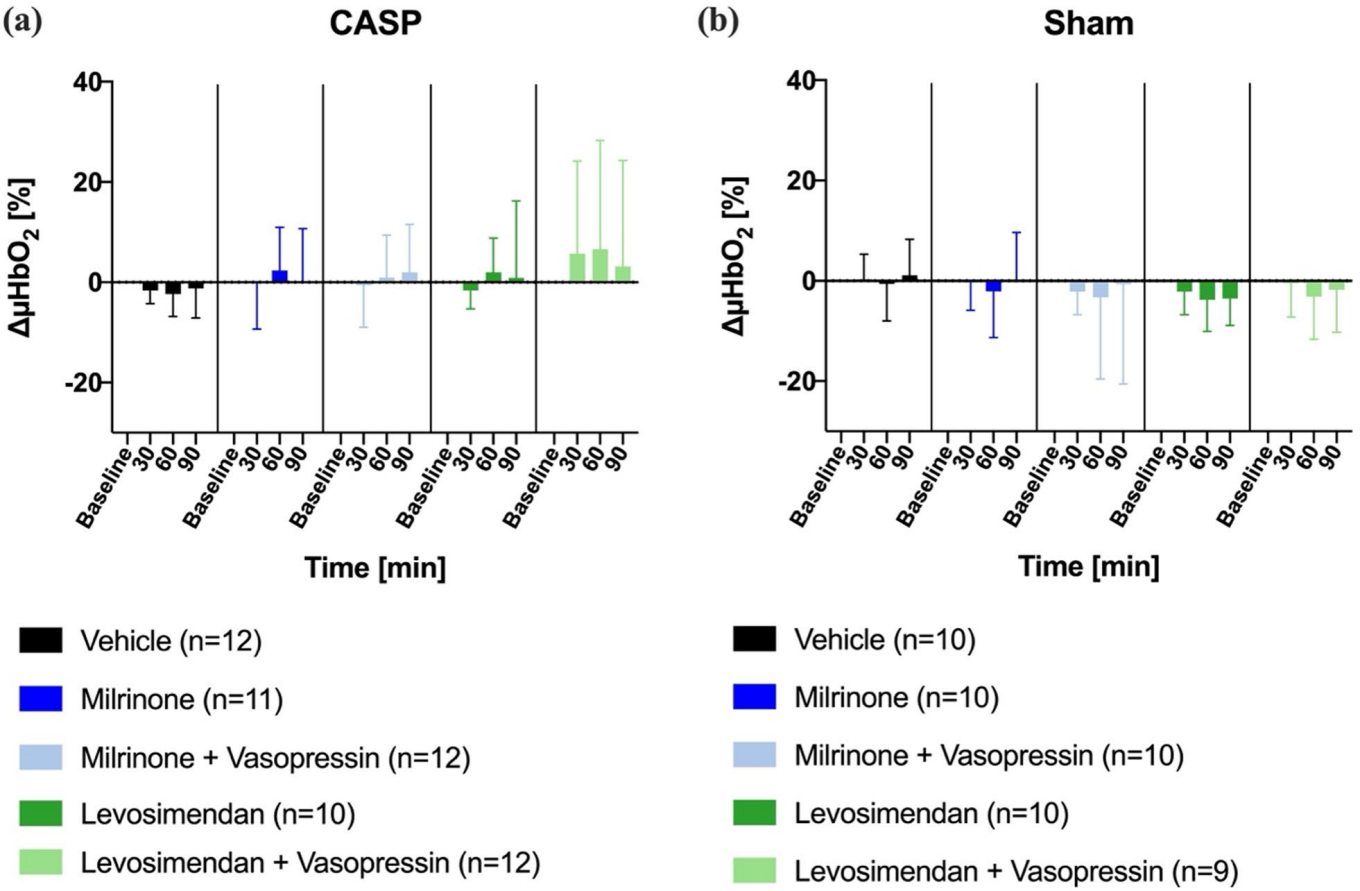
Colonic microvascular oxygenation. Effect of inotrope therapy and vasopressin on microvascular oxygenation (ΔμHbO_2_ [%]) in colon ascendens stent peritonitis (CASP, **a**) or sham (**b**) operated animals. ΔμHbO_2_ values are expressed as changes from the individual baseline. Data are presented as mean ± SD, *n* = 9–12, * = *p* < 0.05 vs. baseline (mixed-effects model with Dunnett post hoc correction)

A pharmacological treatment of septic animals with either milrinone or levosimendan had no effect on colonic µHbO_2_ irrespective of adjunctive vasopressin application (Fig. 3a). Similarly, there was no significant change in colonic µHbO_2_ over time in sham animals (Fig. 3b).

**Fig. 4:**
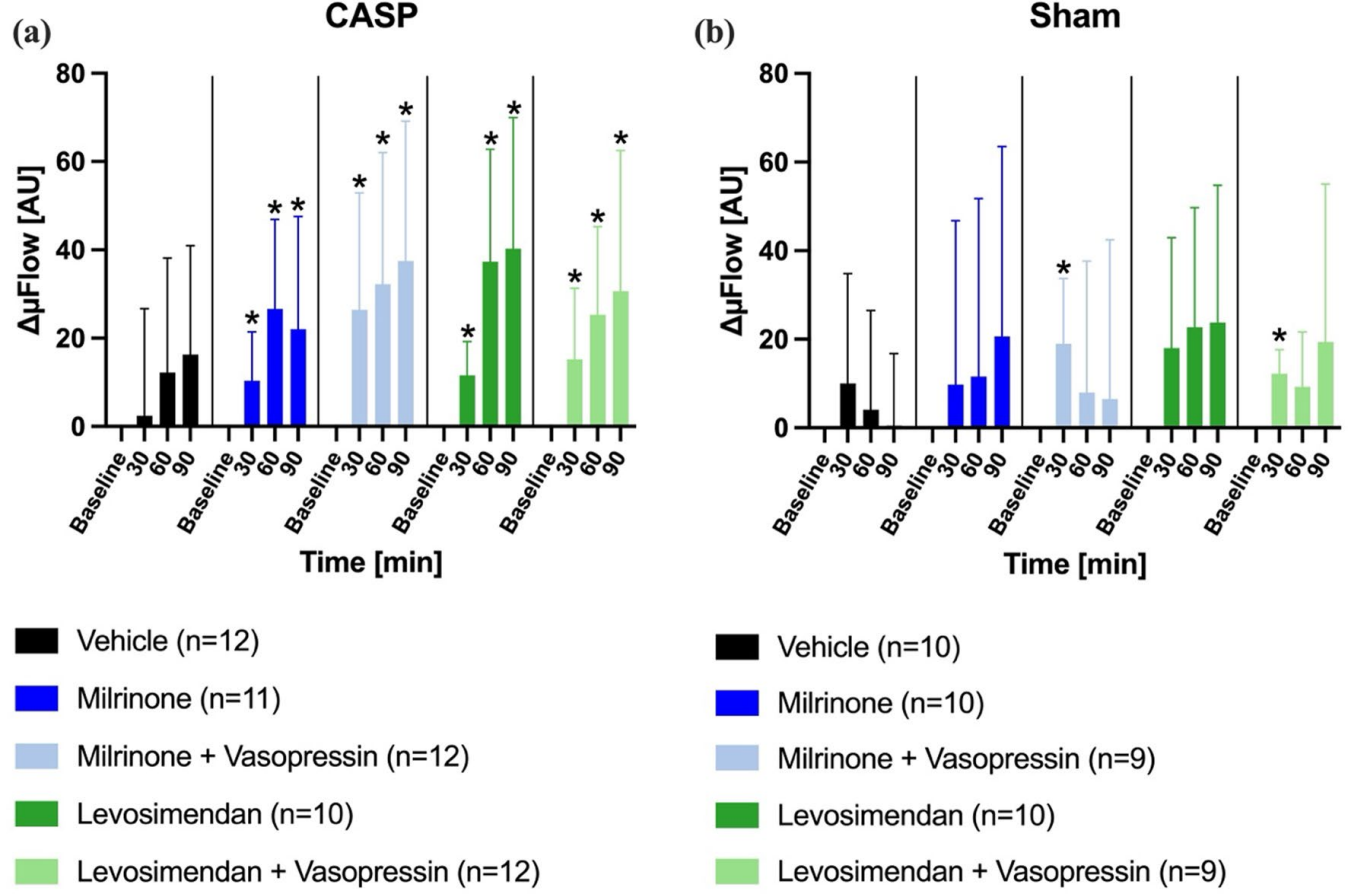
Colonic microvascular blood flow. Effect of inotrope therapy and vasopressin on microvascular blood flow (ΔμFlow [AU]) in colon ascendens stent peritonitis (CASP, **a**) or sham (**b**) operated animals. ΔμFlow values are expressed as changes from the individual baseline. Data are presented as mean ± SD, *n* = 9–12, * = *p* < 0.05 vs. baseline (mixed-effects model with Dunnett post hoc correction)

Microvascular blood flow (µFlow) increased in septic animals following the administration of milrinone, levosimendan, and the combination of each drug with sub-therapeutic vasopressin. In the control group, no statistically significant changes in µFlow were observed (Fig. 4a). In sham-operated animals, a temporary increase in µFlow was detected in the milrinone + vasopressin and levosimendan + vasopressin groups after 30 min (Fig. 4b).

#### Liver

**Fig. 5:**
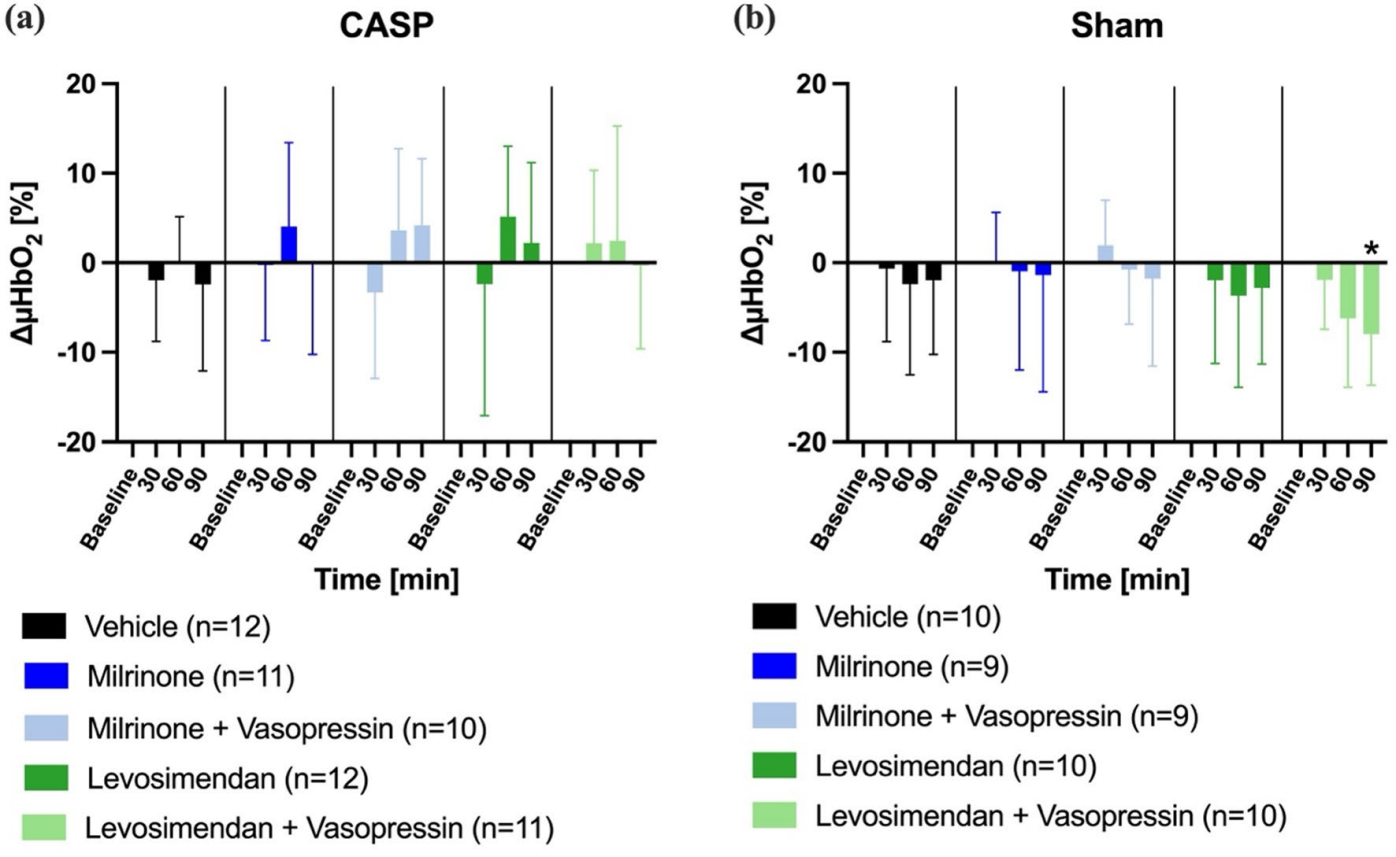
Hepatic microvascular oxygenation. Effect of inotrope therapy and vasopressin on microvascular oxygenation (ΔμHbO_2_ [%]) in colon ascendens stent peritonitis (CASP, **a**) or sham (**b**) operated animals. ΔμHbO_2_ values are expressed as changes from the individual baseline. Data are presented as mean ± SD, *n* = 9–12, * = *p* < 0.05 vs. baseline (mixed-effects model with Dunnett post hoc correction)

In accordance with colonic microcirculatory measurements, hepatic µHbO_2_ did not change over time in septic rats (Fig. 5a). In sham-operated animals, the combination of levosimendan and vasopressin decreased hepatic µHbO_2_, whereas all other treatment groups remained unchanged (Fig. 5b).

**Fig. 6:**
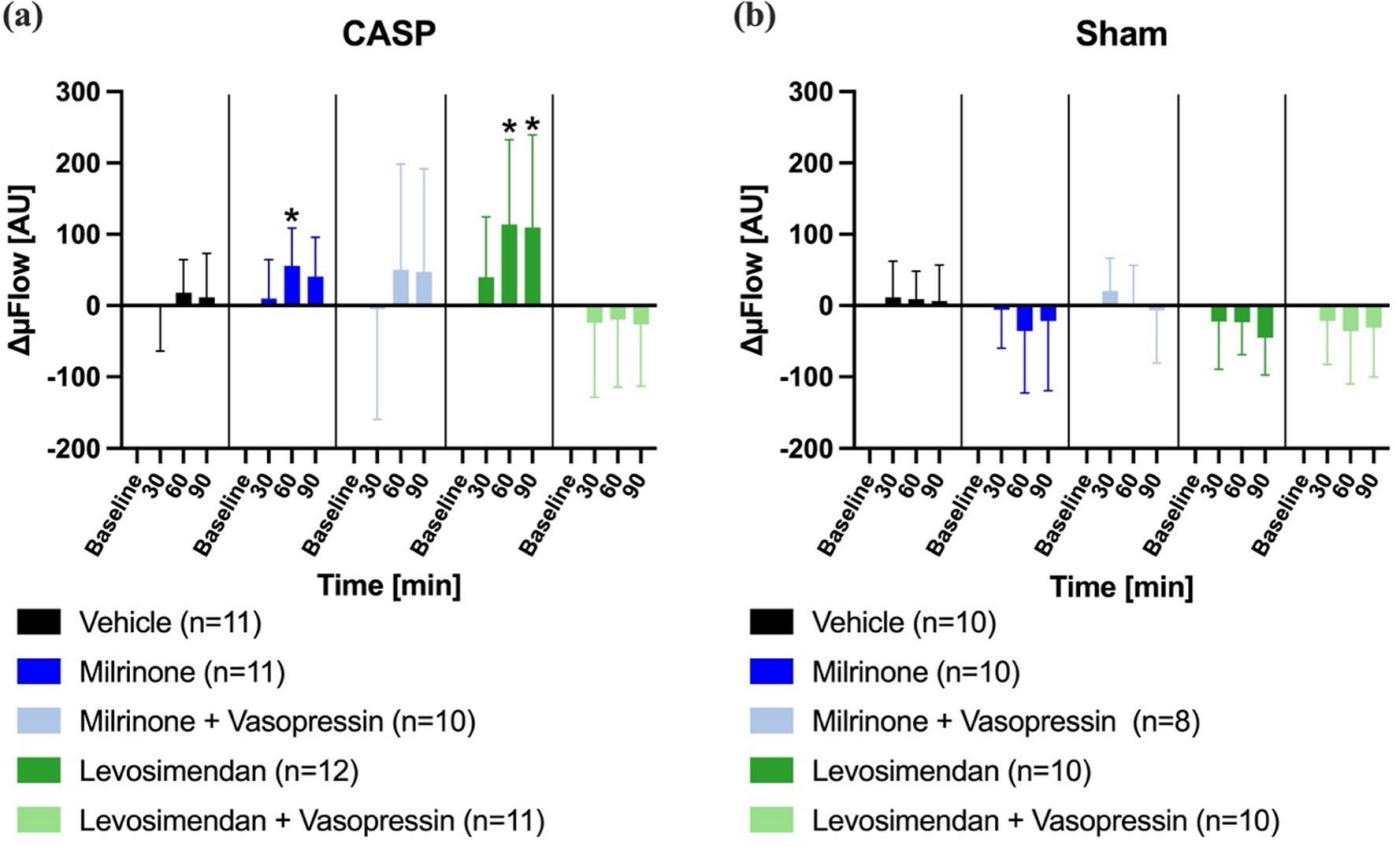
Hepatic microvascular blood flow. Effect of inotrope therapy and vasopressin on microvascular blood flow (ΔμFlow [AU]) in colon ascendens stent peritonitis (CASP, **a**) or sham (**b**) operated animals. ΔμFlow values are expressed as changes from the individual baseline. Data are presented as mean ± SD, *n* = 9–12, * = *p* < 0.05 vs. baseline (mixed-effects model with Dunnett post hoc correction)

Both the milrinone and levosimendan groups revealed an increase of µFlow in the course of the experiments. These effects were not detectable when vasopressin was administered simultaneously (Fig. 6a). In sham animals, µFlow remained unchanged throughout the 90-min observation period (Fig. 6b).

### Mitochondrial respiration

The administration of milrinone and levosimendan with and without vasopressin did not affect colonic mitochondrial respiration after stimulation of the respiratory chain through complex I or II in septic animals. Further, the respiratory control index (RCI) and oxidative phosphorylation efficacy (ADP/O) remained unchanged in all experimental groups. In accordance, pharmacological treatment did not influence hepatic mitochondrial function (Supplemental figure S1 + S2).

## Discussion

The aim of this study was to investigate the effects of commonly used and clinically relevant inotropic agents with and without adjunct sub-therapeutic vasopressin application on intestinal and hepatic microvascular blood flow and oxygenation in septic rats. We further assessed indices of mitochondrial respiration and systemic hemodynamic variables to put microcirculatory measurements into a hemodynamic and metabolic context.

Our main findings are:

1. Milrinone and levosimendan increased colonic and hepatic microvascular blood flow in septic but not in sham-operated rats, without improving microvascular oxygenation.
2. Whereas positively inotropic agents enhanced microvascular blood flow in the liver, the addition of low-dose vasopressin attenuated this effect. In contrast, colonic microvascular blood flow improved both with inotropic therapy alone and with combined inotropic and vasopressin treatment.
3. Mitochondrial function remained unaffected by inotrope therapy across all treatment groups.

Systemic hemodynamic changes observed in this study were consistent with the pharmacologic profiles of the agents used. Both milrinone and levosimendan are inodilators with combined inotropic and vasodilatory properties, and modest alterations in heart rate and mean arterial pressure (MAP) were therefore expected. The transient MAP decline shortly after anesthesia and surgical preparation likely reflects combined effects of anesthesia and buprenorphine [26]. While inotropic therapy attenuated this early drop, MAP remained lower in septic animals treated with milrinone or levosimendan, probably due to peripheral vasodilation that could not be counteracted by the sub-therapeutic vasopressin dose (13.5 mU·kg⁻^1^·h⁻^1^), known to lack pressor activity in rats [38].

In septic animals, microvascular blood flow increased after the application of inotropic agents in particular when measured at the peritoneal surface of the colon, an organ without a pronounced vascular autoregulation or dual blood supply. This confirms our hypothesis that inodilators can improve microvascular perfusion and aligns with the concept of recruiting the microcirculation under adverse conditions using vasodilatory agents. [39, 40]. The less pronounced effects in sham-operated animals, with transient improvements after milrinone or levosimendan application further emphasize the context-specific benefit under septic conditions [41].

However, we want to expose that due to the measurement modality we used to assess microvascular oxygenation and microvascular blood flow we were unable to perform multiple measurements to address intra-organ blood flow heterogeneity which is reported to be one of the mechanisms leading to microcirculatory dysfunction in septic patients. Further, improved microvascular blood flow did not result in improved microvascular oxygenation in the liver and colon. While vasoactive therapy increased colonic and hepatic microvascular blood flow, this should not be interpreted as evidence of improved organ function. The absence of functional readouts (e.g., gut barrier integrity, bacterial translocation, or hepatic functional outcomes) and the unchanged microvascular oxygenation highlight that augmented perfusion does not necessarily translate into effective tissue oxygenation or preserved cellular function. This dissociation between microvascular blood flow and tissue oxygenation is a hallmark of sepsis-induced microvascular dysfunction. Possible mechanisms include heterogeneous perfusion with ongoing shunting away from nutritive capillaries, structural endothelial and glycocalyx alterations that promote interstitial edema and increase the diffusion distance between capillaries and parenchymal cells, and finally, but less likely, impaired oxygen extraction due to mitochondrial dysfunction [42–44]. Prior studies have similarly shown that enhanced microvascular blood flow does not necessarily translate into improved µHbO₂, as oxygen delivery may exceed local metabolic demand or fail to reach dysfunctional cells [45, 46]. The unchanged µHbO₂ in our experiments therefore likely reflects a preserved microvascular blood flow with an ineffective perfusion pattern, rather than insufficient oxygen extraction. However, definitive discrimination between maldistributed flow leading to microvascular shunting and impaired oxygen extraction is not possible based on the present data, as complementary measurements such as local tissue oxygen tension, which provide information beyond microvascular hemoglobin oxygenation, were not part of the experimental design.

Interestingly, in sham-operated animals, the combination of levosimendan and vasopressin reduced hepatic µHbO_2_, suggesting that sub-therapeutic vasopressin may modulate hepatic oxygen availability in the absence of sepsis. This highlights the delicate regulation of microvascular blood flow and underscores the need to understand the concept of context-specific microvascular derangement.

The hepatic microcirculatory findings differed from those observed in the colon. While both milrinone and levosimendan alone increased hepatic microvascular flow, the addition of vasopressin attenuated this effect. This organ-specific response is biologically plausible: The liver’s dual perfusion system and the high density of vasopressin V_1a_ receptors within the hepatic vascular architecture cause hepatic arterial and sinusoidal flow particularly susceptible to vasopressin-induced vasoconstriction, even in the sub-therapeutic dose used in this trial. However, this finding should be interpreted with caution, as the present measurements cannot distinguish intrahepatic redistribution or inflow partitioning from a net reduction in nutritive sinusoidal perfusion. In this context, data from an endotoxemia rabbit model demonstrated that vasopressin, compared with norepinephrine, reduced hepatic arterial blood flow in the early phase, likely by altering portal venous inflow and impairing the hepatic arterial buffer response [47]. Such interference with compensatory mechanisms may counteract the inotrope-mediated vasodilation and abolish the beneficial effects of milrinone and levosimendan alone. In contrast, the intestinal microcirculation appears to be less affected by these vasopressin-induced flow alterations, which may explain why the inotrope-associated improvement in colonic microvascular flow remained preserved. Additionally, it needs to be emphasized that the mentioned sub-therapeutic dose of vasopressin was used in these experiments, which is not expected to result in excessive vasoconstriction and increased MAP in rats.

It becomes clear that microvascular alterations and the response to pharmacological and therapeutic approaches are organ-specific.

Mitochondrial respiration remained unchanged in both colonic and hepatic tissue after all interventions in septic animals. One limitation of the assessment of mitochondrial respiration in this study was the use of whole-tissue homogenates normalized to total protein, which may not fully capture differences in mitochondrial content or compartmentalization. However, this approach was intentionally chosen to enable spatial and temporal alignment with measurements of microvascular perfusion and tissue oxygenation, and within these constraints no trend toward altered mitochondrial respiration was observed. This preservation of oxidative capacity, despite microvascular impairment, suggests that mitochondrial function is maintained during the early phase of sepsis. Previous studies have shown that mitochondrial dysfunction evolves heterogeneously and is often preceded by compensatory metabolic adaptation rather than an immediate loss of respiratory capacity [35, 48, 49]. Thus, the preserved mitochondrial respiration observed here is likely to reflect the early and hemodynamically compensated disease stage rather than a true uncoupling between microvascular perfusion and mitochondrial function. Consistent with our prior CASP data indicating compensatory preservation of mitochondrial function in early sepsis, we did not expect deterioration but examined whether vasopressors and inodilators could further augment this response [48]. As outlined in the introduction section, this study aimed to investigate pharmacological effects of levosimendan, milrinone and vasopressin in this early disease state, since the absence of adequate organ replacement therapies for the gastrointestinal tract and the liver places particular emphasis on pharmacological and conceptual preventive strategies in critically ill patients. We therefore interpret our data as indicating preserved oxidative capacity despite impaired oxygen delivery, not mitochondrial insensitivity to microvascular dysfunction. Our trial investigated the effect of inotropic agents on microcirculation and mitochondrial function. The results we measured align with several animal studies utilizing milrinone and levosimendan in canine, porcine, rat and hamster models [19, 50–52] as well as two randomized controlled clinical trials utilizing sublingual microcirculatory measurements [53, 54]. In contrast, one experimental study in 40 rats using sidestream darkfield imaging of the buccal mucosa after cecal ligation and puncture did not demonstrate an improvement in microvascular perfusion with levosimendan, despite restoration of cardiac output [55]. However, several important differences may explain this apparent discrepancy. First, this study assessed buccal rather than splanchnic microcirculation, which, according to our results, may behave differently in sepsis. Second, it focused on a different phase of septic shock, applied another levosimendan dosing regimen, and used alternative endpoints to define microvascular perfusion. Interestingly, levosimendan improved microvascular oxygenation without changing perfusion, suggesting tissue-specific or metabolic effects that cannot be directly extrapolated to abdominal organ beds. To our knowledge, no previous study has combined an inodilator with sub-therapeutic vasopressin to examine splanchnic microcirculation in sepsis. Our work therefore extends the existing evidence by highlighting potential organ-specific interactions between vasodilatory inotropes and vasoconstrictive adjuncts at the microvascular level.

One of the strengths of our study is the model we choose. The colon ascendens stent peritonitis which is an alternate model to the gold standard cecal ligation and puncture (CLP) [56]. We decided to use this model based on three reasons: First, our long time experience with this model, with which we conducted several studies over the last years, modifying the number and size of stents, leading to a good balance of the severity of sepsis, resulting in a moderate sepsis, without sepsis-related mortality and development of septic shock [57, 58]. Accordingly, our findings primarily apply to the early, hemodynamically compensated phase of abdominal sepsis. While this limits direct extrapolation to advanced septic shock with vasopressor dependence, it allows us to isolate microcirculatory and mitochondrial drug effects without confounding by profound hypotension, high catecholamine exposure, or multi-organ failure. However, further studies should investigate the effect of the here used drugs on microcirculatory and mitochondrial function in models of more severe sepsis. Second, the model is widely evaluated by many groups in terms of cytokines and other inflammatory markers and sufficiently resembles the course of sepsis [59–61]. Third, the way of implementing sepsis by a stent is the closest to mimic the clinical way an abdominal sepsis develops e.g. through a perforation or anastomotic insufficiency. Another major strength is the rigorous study design: animals were randomly allocated to treatment groups in a randomized, placebo-controlled, blinded fashion, with predefined CASP (sepsis) and sham (laparotomy) groups, enabling a clear distinction between septic and non-septic conditions and minimizing selection and observer bias. In addition, we applied a multimodal assessment strategy combining systemic hemodynamics, intestinal and hepatic microvascular perfusion and oxygenation, and mitochondrial respiration, allowing an integrated evaluation of drug effects across macro-, micro- and cellular levels.

However, our study has several limitations. First, unequal group sizes required the use of mixed-effects models. The deliberate reduction of sham-operated animals in line with the 3R principle may have reduced statistical power in the control groups and increased the risk of type I or type II error for some comparisons. Second, we used only male rats. This choice was made to account for known sex- and hormone-dependent differences in intestinal barrier properties and inflammatory responses, and to ensure a homogeneous model. However, a growing body of evidence indicates that sepsis-associated microcirculatory dysfunction and pharmacologic responses differ between sexes, influenced by sex hormones, endothelial signaling, and immune–vascular interactions [62]. Accordingly, the translational generalizability of our findings to both sexes in human sepsis is limited. Third, we did not measure advanced cardiovascular parameters such as cardiac output, cardiac index, or systemic vascular resistance. These variables would have allowed a more comprehensive characterization of the inotropic and vasodilatory effects of milrinone and levosimendan and their interaction with vasopressin. For example, an increase in cardiac output may increase systemic perfusion, potentially improving microvascular flow independently of local vasomotor regulation. Conversely, systemic vasodilation with redistribution of flow could exacerbate microvascular heterogeneity, particularly in organs with complex autoregulatory mechanisms. These effects may differ between agents, as milrinone predominantly reduces afterload via phosphodiesterase-III inhibition, whereas levosimendan additionally enhances myocardial efficiency and may exert more pronounced microvascular vasodilation through potassium-ATP channel activation.

Despite these limitations, our results may have implications for future hemodynamic management in sepsis with septic cardiomyopathy and microcirculatory impairment. Inodilators such as milrinone or levosimendan could be a useful agent in restoring microvascular perfusion in selected patients with preserved blood pressure but impaired microvascular blood flow. However, organ-specific responses as observed in the liver highlight the need for individualized hemodynamic strategies and caution against indiscriminate vasopressin use in early sepsis resuscitation.

## Summary

In summary, this experimental study demonstrates that inodilator therapy with milrinone and levosimendan improves microvascular blood flow in sepsis, even in the absence of major systemic hemodynamic changes. However, these flow improvements did not translate into higher microvascular oxygenation. The addition of sub-therapeutic vasopressin had organ-specific effects: neutral in the colon but antagonistic in the liver, highlighting the complex interplay between vasodilatory and vasoconstrictive mechanisms across different vascular beds. Mitochondrial respiration remained unaltered, suggesting that early alterations during moderate sepsis are primarily microvascular rather than mitochondrial. Together, these findings emphasize that targeting microcirculatory function may offer additional therapeutic potential in sepsis beyond conventional systemic hemodynamic optimization.

## Supplementary Information

Additional file 1

## Data Availability

The datasets used and analysed during the current study are available from the corresponding author on reasonable request.

## Abbreviations

ADP/O: Adenosine diphosphate to oxygen ratio
ATP: Adenosine triphosphate
ANOVA: Analysis of variance
AU: Arbitrary units
CASP: Colon ascendens stent peritonitis
FiO₂: Fraction of inspired oxygen
FiN₂: Fraction of inspired nitrogen
HR: Heart rate
i.p.: Intraperitoneal
i.v.: Intravenous
MAP: Mean arterial pressure
MODS: Multiple organ dysfunction syndrome
PAW: Airway pressure
RCI: Respiratory control index
SD: Standard deviation
SOFA: Sequential Organ Failure Assessment
SRSS: Septic Rat Severity Score
VT: Tidal volume
µFlow: Microvascular blood flow
µHbO₂: Microvascular hemoglobin oxygenation
