# Supplementary material for: Milrinone and levosimendan improve microvascular perfusion in septic rats: a randomized, placebo-controlled trial
Source: Intensive Care Med Exp. 2026 Mar 6;14:30. doi: 10.1186/s40635-026-00881-w (PMC12965941; doi:10.1186/s40635-026-00881-w)
Supplement: Supplementary file 1 — Additional file 1 [file 40635_2026_881_MOESM1_ESM.docx]

**Supplement: Milrinone and levosimendan improve microvascular perfusion in septic rats - A randomized, placebo-controlled trial**

**Figure S1: Mitochondrial measurements in colon homogenates**


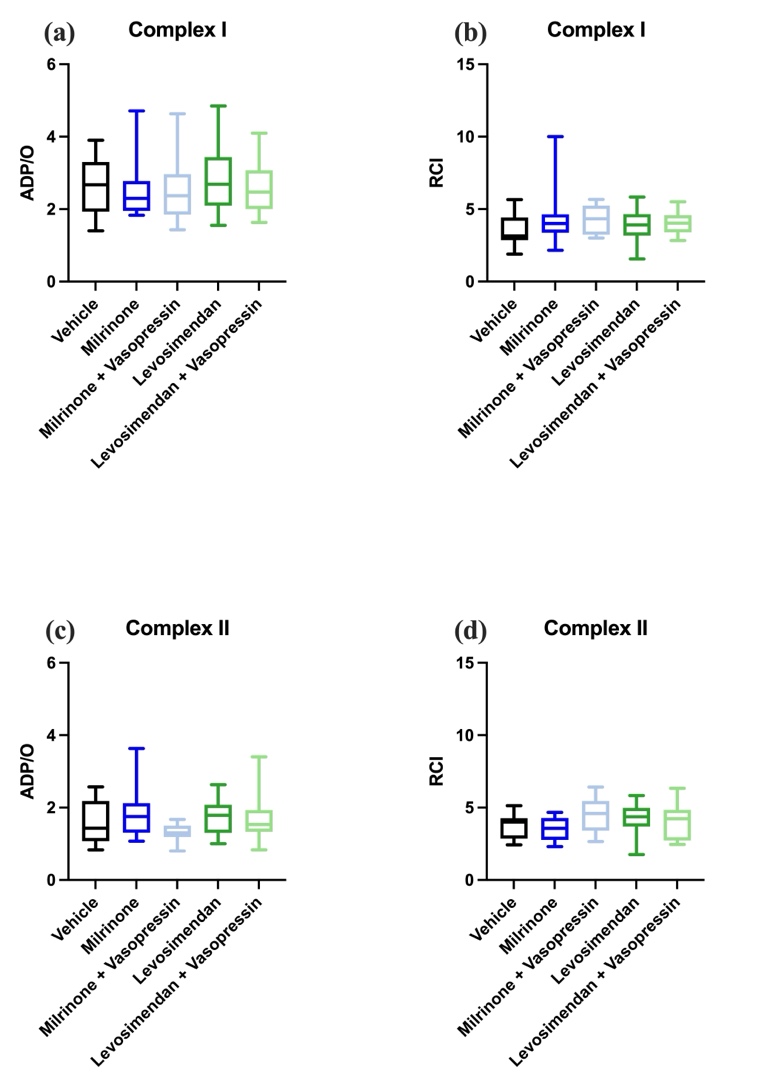


Effect of inotropes and vasopressin on respiration expressed as ADP/O (a,c) and RCI (b,d) after stimulation of the respiratory chain through complex I (a,b) and II (c,d) in colon under septic conditions. Data are presented as min/median/max, n = 10 per group, Kruskal–Wallis test with Dunn’s post hoc correction for multiple comparisons.

**Figure S2: Mitochondrial measurements in liver homogenates**


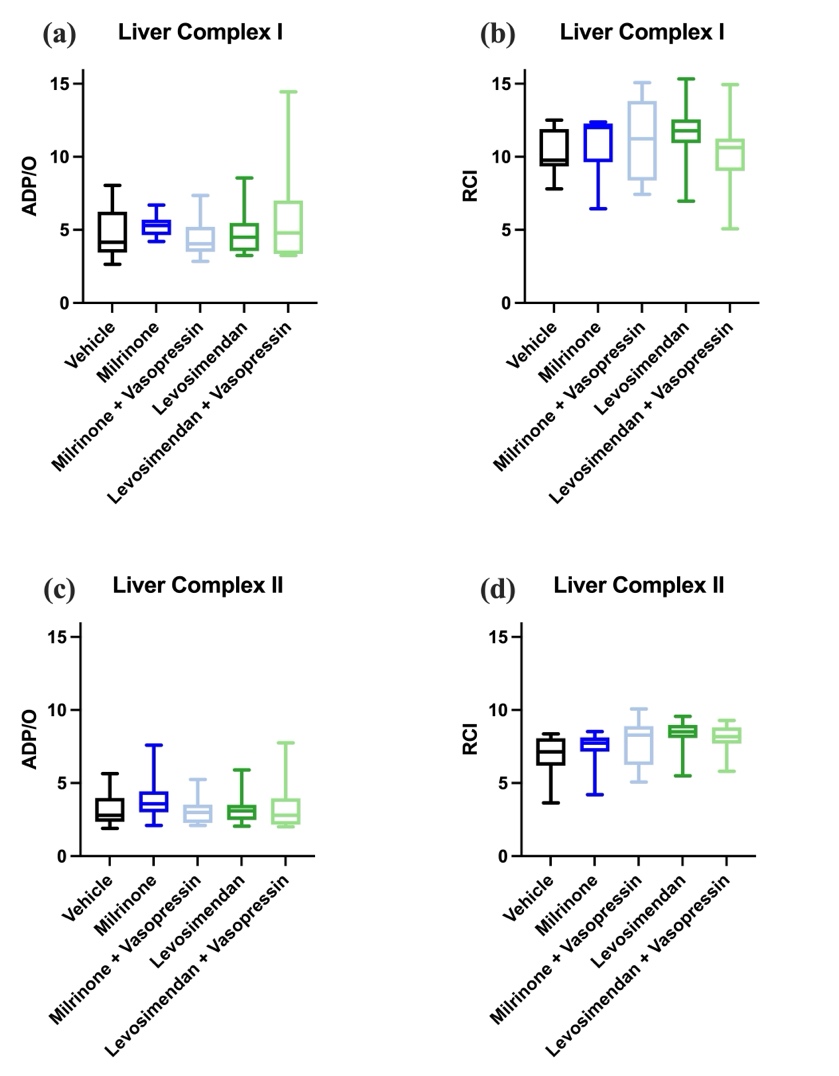


Effect of inotropes and vasopressin on respiration expressed as ADP/O (a,c) and RCI (b,d) after stimulation of the respiratory chain through complex I (a,b) and II (c,d) in colon under septic conditions. Data are presented as min/median/max, n = 10 per group, Kruskal–Wallis test with Dunn’s post hoc correction for multiple comparisons.

**Table S3: Systemic vital parameters**

| **Time [min]** | **Mean** | **SD** | **n** | **Mean** | **SD** | **n** | **Mean** | **SD** | **n** | **Mean** | **SD** | **n** | **Mean** | **SD** | **n** |
| --- | --- | --- | --- | --- | --- | --- | --- | --- | --- | --- | --- | --- | --- | --- | --- |
| **CASP HR [bpm]** | Vehicle | | | Milrinon | | | Levosimendan | | | Milrinon +  Vasopressin | | | Levosimendan + Vasopressin | | |
| 0 | 470 | 54 | 11 | 471 | 35 | 12 | 456 | 43 | 12 | 486 | 32 | 12 | 449 | 46 | 12 |
| 30 | 450 | 61 | 11 | 464 | 43 | 12 | 451 | 43 | 12 | 482 | 36 | 12 | 446 | 46 | 12 |
| 60 | 439 | 62 | 11 | 457 | 36 | 12 | 447 | 53 | 12 | 469 | 42 | 12 | 449 | 48 | 12 |
| 90 | 429 | 64 | 11 | 457 | 41 | 12 | 450 | 52 | 12 | 475 | 51 | 12 | 458 | 43 | 12 |
|  | | | | | | | | | | | | | | | |
| **Sham HR [bpm]** | Vehicle | | | Milrinon | | | Levosimendan | | | Milrinon +  Vasopressin | | | Levosimendan + Vasopressin | | |
| 0 | 443 | 40 | 10 | 447 | 45 | 10 | 470 | 48 | 10 | 446 | 41 | 9 | 456 | 46 | 10 |
| 30 | 431 | 62 | 10 | 437 | 52 | 10 | 469 | 50 | 10 | 450 | 48 | 9 | 442 | 45 | 10 |
| 60 | 415 | 57 | 10 | 429 | 56 | 10 | 470 | 44 | 10 | 441 | 47 | 9 | 435 | 44 | 10 |
| 90 | 419 | 51 | 10 | 440 | 60 | 10 | 477 | 29 | 10 | 443 | 46 | 9 | 439 | 46 | 10 |
|  | | | | | | | | | | | | | | | |
| **CASP MAP [mmHg]** | Vehicle | | | Milrinon | | | Levosimendan | | | Milrinon +  Vasopressin | | | Levosimendan + Vasopressin | | |
| 0 | 97 | 20 | 12 | 106 | 18 | 12 | 111 | 15 | 12 | 110 | 25 | 12 | 111 | 22 | 12 |
| 30 | 82 | 23 | 12 | 78 | 17 | 12 | 81 | 18 | 12 | 91 | 23 | 12 | 87 | 35 | 12 |
| 60 | 89 | 25 | 12 | 91 | 20 | 12 | 87 | 14 | 12 | 92 | 21 | 12 | 87 | 31 | 12 |
| 90 | 86 | 25 | 12 | 85 | 17 | 12 | 92 | 17 | 12 | 97 | 20 | 12 | 90 | 32 | 12 |
|  | | | | | | | | | | | | | | | |
| **Sham MAP [mmHg]** | Vehicle | | | Milrinon | | | Levosimendan | | | Milrinon +  Vasopressin | | | Levosimendan + Vasopressin | | |
| 0 | 117 | 29 | 10 | 110 | 24 | 10 | 115 | 29 | 10 | 117 | 15 | 9 | 124 | 16 | 10 |
| 30 | 107 | 30 | 10 | 93 | 26 | 10 | 95 | 20 | 10 | 93 | 8 | 9 | 103 | 20 | 10 |
| 60 | 100 | 27 | 10 | 92 | 23 | 10 | 90 | 19 | 10 | 95 | 9 | 9 | 102 | 24 | 10 |
| 90 | 106 | 33 | 10 | 101 | 17 | 10 | 99 | 23 | 10 | 100 | 11 | 9 | 106 | 25 | 10 |

Abbreviations: CASP: Colon ascendes stent peritonitis, SD: Standard deviation, n: Number of animals per group

**Table S4: Microcirculatory values in colon**

| **Colon** | **Mean** | **SD** | **n** | **Mean** | **SD** | **n** | **Mean** | **SD** | **n** | **Mean** | **SD** | **n** | **Mean** | **SD** | **n** |
| --- | --- | --- | --- | --- | --- | --- | --- | --- | --- | --- | --- | --- | --- | --- | --- |
| **CASP ΔµHbO2 [%]** | Vehicle | | | Milrinon | | | Levosimendan | | | Milrinon +  Vasopressin | | | Levosimendan + Vasopressin | | |
| 0 | 0.0 | 0.0 | 12 | 0.0 | 0.0 | 11 | 0.0 | 0.0 | 10 | 0.0 | 0.0 | 12 | 0.0 | 0.0 | 12 |
| 30 | -1.6 | 2.6 | 12 | -0.1 | 9.2 | 11 | -1.6 | 3.7 | 10 | -0.6 | 8.4 | 12 | 5.7 | 18.4 | 12 |
| 60 | -2.3 | 4.5 | 12 | 2.3 | 8.7 | 11 | 2.0 | 6.8 | 10 | 0.9 | 8.4 | 12 | 6.6 | 21.7 | 12 |
| 90 | -1.2 | 5.9 | 12 | 0.0 | 10.7 | 11 | 0.9 | 15.4 | 10 | 2.0 | 9.5 | 12 | 3.2 | 21.1 | 12 |
|  | | | | | | | | | | | | | | | |
| **Sham ΔµHbO2 [%]** | Vehicle | | | Milrinon | | | Levosimendan | | | Milrinon +  Vasopressin | | | Levosimendan + Vasopressin | | |
| 0 | 0.0 | 0.0 | 10 | 0.0 | 0.0 | 10 | 0.0 | 0.0 | 10 | 0.0 | 0.0 | 10 | 0.0 | 0.0 | 9 |
| 30 | 0.3 | 5.0 | 10 | -0.3 | 5.6 | 10 | -2.1 | 4.6 | 10 | -2.2 | 4.6 | 10 | -0.5 | 6.7 | 9 |
| 60 | -0.6 | 7.4 | 10 | -2.1 | 9.3 | 10 | -3.8 | 6.3 | 10 | -3.3 | 16.3 | 10 | -3.1 | 8.5 | 9 |
| 90 | 1.1 | 7.2 | 10 | 0.2 | 9.4 | 10 | -3.5 | 5.4 | 10 | -0.7 | 19.9 | 10 | -1.8 | 8.5 | 9 |
|  | | | | | | | | | | | | | | | |
| **CASP  ΔµFlow [AU]** | Vehicle | | | Milrinon | | | Levosimendan | | | Milrinon +  Vasopressin | | | Levosimendan + Vasopressin | | |
| 0 | 0.0 | 0.0 | 12 | 0.0 | 0.0 | 11 | 0.0 | 0.0 | 10 | 0.0 | 0.0 | 12 | 0.0 | 0.0 | 12 |
| 30 | 2.4 | 24.3 | 12 | 10.4 | 11.0 | 11 | 11.6 | 7.6 | 10 | 26.4 | 26.5 | 12 | 15.2 | 16.1 | 12 |
| 60 | 12.3 | 25.9 | 12 | 26.7 | 20.2 | 11 | 37.3 | 25.4 | 10 | 32.2 | 29.8 | 12 | 25.3 | 19.9 | 12 |
| 90 | 16.3 | 24.7 | 12 | 22.1 | 25.5 | 11 | 40.3 | 29.7 | 10 | 37.5 | 31.7 | 12 | 30.6 | 31.9 | 12 |
|  | | | | | | | | | | | | | | | |
| **Sham  ΔµFlow [AU]** | Vehicle | | | Milrinon | | | Levosimendan | | | Milrinon +  Vasopressin | | | Levosimendan + Vasopressin | | |
| 0 | 0.0 | 0.0 | 10 | 0.0 | 0.0 | 10 | 0.0 | 0.0 | 10 | 0.0 | 0.0 | 9 | 0.0 | 0.0 | 9 |
| 30 | 10.0 | 24.8 | 10 | 9.7 | 37.0 | 10 | 18.1 | 24.8 | 10 | 19.0 | 14.7 | 9 | 12.2 | 5.4 | 9 |
| 60 | 4.1 | 22.4 | 10 | 11.6 | 40.2 | 10 | 22.7 | 27.0 | 10 | 8.0 | 29.6 | 9 | 9.3 | 12.4 | 9 |
| 90 | 0.5 | 16.3 | 10 | 20.6 | 42.9 | 10 | 23.8 | 31.0 | 10 | 6.5 | 36.0 | 9 | 19.3 | 35.7 | 9 |

Abbreviations: CASP: Colon ascendes stent peritonitis, SD: Standard deviation, n: Number of animals per group

**Table S5: Microcirculatory values in liver**

| **Liver** | **Mean** | **SD** | **n** | **Mean** | **SD** | **n** | **Mean** | **SD** | **n** | **Mean** | **SD** | **n** | **Mean** | **SD** | **n** |
| --- | --- | --- | --- | --- | --- | --- | --- | --- | --- | --- | --- | --- | --- | --- | --- |
| **CASP ΔµHbO2 [%]** | Vehicle | | | Milrinon | | | Levosimendan | | | Milrinon + Vasopressin | | | Levosimendan + Vasopressin | | |
| 0 | 0.0 | 0.0 | 12 | 0.0 | 0.0 | 11 | 0.0 | 0.0 | 12 | 0.0 | 0.0 | 10 | 0.0 | 0.0 | 11 |
| 30 | -2.0 | 6.8 | 12 | -0.2 | 8.4 | 11 | -2.4 | 14.7 | 12 | -3.3 | 9.6 | 10 | 2.2 | 8.1 | 11 |
| 60 | 0.2 | 5.0 | 12 | 4.0 | 9.4 | 11 | 5.2 | 7.9 | 12 | 3.6 | 9.1 | 10 | 2.4 | 12.9 | 11 |
| 90 | -2.4 | 9.7 | 12 | -0.1 | 10.1 | 11 | 2.2 | 9.0 | 12 | 4.2 | 7.4 | 10 | -0.3 | 9.3 | 11 |
|  | | | | | | | | | | | | | | | |
| **Sham ΔµHbO2 [%]** | Vehicle | | | Milrinon | | | Levosimendan | | | Milrinon + Vasopressin | | | Levosimendan + Vasopressin | | |
| 0 | 0.0 | 0.0 | 10 | 0.0 | 0.0 | 9 | 0.0 | 0.0 | 10 | 0.0 | 0.0 | 9 | 0.0 | 0.0 | 10 |
| 30 | -0.6 | 8.2 | 10 | 0.1 | 5.5 | 9 | -2.0 | 9.3 | 10 | 2.0 | 5.0 | 9 | -1.9 | 5.6 | 10 |
| 60 | -2.4 | 10.1 | 10 | -1.0 | 11.0 | 9 | -3.7 | 10.3 | 10 | -0.7 | 6.1 | 9 | -6.2 | 7.7 | 10 |
| 90 | -1.9 | 8.3 | 10 | -1.4 | 13.0 | 9 | -2.8 | 8.5 | 10 | -1.8 | 9.8 | 9 | -8.0 | 5.7 | 10 |
|  | | | | | | | | | | | | | | | |
| **CASP  ΔµFlow [AU]** | Vehicle | | | Milrinon | | | Levosimendan | | | Milrinon + Vasopressin | | | Levosimendan + Vasopressin | | |
| 0 | 0.0 | 0.0 | 11 | 0.0 | 0.0 | 11 | 0.0 | 0.0 | 12 | 0.0 | 0.0 | 10 | 0.0 | 0.0 | 11 |
| 30 | -0.8 | 63.2 | 11 | 9.7 | 54.8 | 11 | 39.7 | 84.6 | 12 | -5.1 | 154.7 | 10 | -24.4 | 104.1 | 11 |
| 60 | 18.2 | 46.3 | 11 | 55.5 | 53.1 | 11 | 113.9 | 118.9 | 12 | 50.2 | 148.4 | 10 | -19.7 | 94.7 | 11 |
| 90 | 11.6 | 61.7 | 11 | 40.6 | 55.6 | 11 | 109.4 | 130.0 | 12 | 47.2 | 144.6 | 10 | -26.6 | 86.4 | 11 |
|  | | | | | | | | | | | | | | | |
| **Sham  ΔµFlow [AU]** | Vehicle | | | Milrinon | | | Levosimendan | | | Milrinon +  Vasopressin | | | Levosimendan + Vasopressin | | |
| 0 | 0.0 | 0.0 | 10 | 0.0 | 0.0 | 10 | 0.0 | 0.0 | 10 | 0.0 | 0.0 | 8 | 0.0 | 0.0 | 10 |
| 30 | 11.4 | 50.7 | 10 | -6.0 | 53.8 | 10 | -22.2 | 67.2 | 10 | 20.5 | 46.1 | 8 | -21.5 | 61.1 | 10 |
| 60 | 9.1 | 39.1 | 10 | -35.6 | 87.2 | 10 | -23.5 | 45.4 | 10 | 2.5 | 53.8 | 8 | -36.1 | 73.9 | 10 |
| 90 | 6.5 | 50.6 | 10 | -21.4 | 97.8 | 10 | -45.2 | 52.0 | 10 | -7.1 | 73.6 | 8 | -31.1 | 69.0 | 10 |

Abbreviations: CASP: Colon ascendes stent peritonitis, SD: Standard deviation, n: Number of animals per group
